# Supplementary material for: Antimalarial target vulnerability of the putative Plasmodium falciparum methionine synthase
Source: PeerJ. 2024 Jan 15;12:e16595. doi: 10.7717/peerj.16595 (PMC10795524; doi:10.7717/peerj.16595)
Supplement: Supplemental Information 1 — Protein sequences were obtained from UniProt of the cobalamin-dependent methionine synthase (MS) enzymes from human (MTR, Q99707-1) and Escherichia coli K12 (metH, P13009), together with Plasmodium candidate MS from orthologous group 1324at5820 encoded by P. falciparum PF3D7_1233700 (Pf, Q8I585), P. knowlesi PKH_145080 (Pk, A0A384KWI2), P. malariae PmUG01_14067900 (Pm, A0A1A8X239), P. ovale wallikeri PowCR01_140053700 (Pow, A0A1C3L5P3), P. ovale curtisi PocGH01_14059300 (Poc, A0A1D3UAH7), and P. vivax PVX_100640 (Pv, A0A1G4H5F2) genes. Sequences were aligned using the T-Coffee tool with default options in the Expresso web interface (Armougom et al., 2006). [file peerj-12-16595-s001.pdf]

```
T-COFFEE, Version_11.00 (Version_11.00)
Cedric Notredame
CPU TIME:0 sec.
SCORE=777
*
 BAD AVG GOOD
*
PF3D7_1233700      :  80
PKH_145080         :  82
PmUG01_14067900    :  81
PowCR01_1400537    :  82
PocGH01_1405930    :  82
PVX_100640         :  83
metH               :  63
MTR                :  67
cons               :  77

PF3D7_1233700      -------------------------MRKDLYTLDGGKISELGRLGYENFDFLSY-----EMNE----EN--ENNDN-MISILEN
PKH_145080         -------------------------MAKEVFTLDGGKISEFERLGLGNFDFLSY-----PQ------ECN---KEE-MVSTLES
PmUG01_14067900    MDK----------------------MAKHIYTLDGGKITELERMGFRNFDFLSC-----EDNEEKEEEGGTEKKEM-MMQILEN
PowCR01_1400537    -------------------------MRKQIHTLDGGKISELERLGFESFDFLSL-----EEGK----EGDTEKMEE-MKKGLEN
PocGH01_1405930    -------------------------MRKQIHTLDGGKISELERLGFETFDFLSL-----EEGK----EGNTEKMEE-MKKGLEN
PVX_100640         -------------------------MAKEVFTLDGGKISELERLGVGNFDFLSC-----HD------EGD---EEQ-MVSTLEN
metH               MSSKVEQL--------------RAQLNERILVLDGGMGTMIQSYRLNEADFRGERFADWPCDL----KGNNDLLVLSKPEVIAA
MTR                MSPALQDLSQPEGLKKTLRDEINAILQKRIMVLDGGMGTMIQREKLNEEHFRGQEFKDHARPL----KGNNDILSITQPDVIYQ

cons                                        : : : .****  : :        .* .              :           . :


PF3D7_1233700      IHLSYLLGGCNIIGTNTFQVNLYSFKK--LGIDNGEEILNKYINIAYNSLLKYEEIKRKSKDD-----------INV-LLNPRN
PKH_145080         IHLSYLLAGCNVISTNTFQVNLHSLQEKGISIEEGQGIIDTYIDIAHNALLKYERIKRTTDFP-----------LHLGMTQVEE
PmUG01_14067900    IHLSYFLAGSDIICTNTFQVNLHSLRKKNISIEKGEEILNTYIDIAYNSAEKYKEIKRKKGNL-----------W----EN-RK
PowCR01_1400537    LHLSYFLAGSNITCTNTFQVNLHSLRERNISIERGNEILDTYIDIAYNSVQRFRDIRRNKNSF-----------I----KWKKC
PocGH01_1405930    LHLSYFLAGSNITCTNTFQVNLHSLRERNISIERGNEILNTYIDIAYNSVRSFRDIRRNNRSF-----------I----KWKKC
PVX_100640         IHLSYLLAGCNVISTNTFQVNLHSLQEKGISVQDGEGIVDRYIDIAHRALLRYEGIKRSEDFP-----------L-FH--LCPL
metH               IHNAYFEAGADIIETNTFNSTTIAMADY--QME----SLSAEINFAAAKLAR------ACADEWTARTPEKPRYVAG-VLGPTN
MTR                IHKEYLLAGADIIETNTFSSTSIAQADY--GLE----HLAYRMNMCSAGVAR------KAAEEVTLQTGI-KRFVAG-ALGPTN

cons               :*  *: .*.::  ****. .  :  .    ::     :   :::.


PF3D7_1233700      KNISVY----------------------------DYLSSYHKIRKKFDILHEKDVSFNIDNYLKDIKIEQ----------N---
PKH_145080         KESSPY----------------------------DHLEKFQKMRIHWGILHPNDS-VDTREYVDRFDLND----------D---
PmUG01_14067900    QNDSIY----------------------------EHLENFHNIRKDLNNVHSTDI-FNVEEYLSELQLND----------T---
PowCR01_1400537    RSKSVN----------------------------DHLIHFQKVRTALNVVHPNDI-FNVSEYMLEFDLID----------R---
PocGH01_1405930    RSKSVN----------------------------DHLIDFQKVRTALNVVHPNDI-FNVSEYMLEFDLVD----------R---
PVX_100640         EDSSPY----------------------------EHLEGFQQMRNHLGIVHPKDS-VNTREYVDSFDLSP----------G---
metH               RTASISPDVNDPAFRNITFDGLVAAYRESTKALVEGGADLILIETVFDTLNAKAAVFAVKTEFEALGVELPIMISGTITDASGR
MTR                KTLSVSPSVERPDYRNITFDELVEAYQEQAKGLLDGGVDILLIETIFDTANAKAALFALQNLFEEKYAPRPIFISGTIVDKSGR

cons               .  *                              :       :.   .  : .   .     .


PF3D7_1233700      -------------------------------IGH--NKYRKFNKN-RN-NYISFSNGPYSSTFADFSEYSGVMQKNKEDKN---
PKH_145080         -------------------------------V----WTGNPIRRC-QD-YYVAFSTGGYSSAFRDFSEYSGVLRKERKATNWG-
PmUG01_14067900    -------------------------------QNEWKWAKTKLERGPKS-CCVAFSNGSYSSAFCDFSEYSGVFHKNRRKKE---
PowCR01_1400537    -------------------------------ENR--KVMEKIRRS-NE-YCVSFSNGPYNSAFRDFSEYSGVLQKNKGPD----
PocGH01_1405930    -------------------------------ENR--TVMEKIRRN-NE-YCVSFSNGPYNSAFRDFSEYSGVLQKYKGLD----
PVX_100640         -------------------------------G----WGGSPIRRC-PG-RYVAFATGGYSAAFRDFSEYSGVMRKGGAPGK---
metH               TLSGQTTEAFYNSLRHAEALTFGLNCALGPDELR--QYVQELSRI-AECYVTAHPNAGLPNAFGEYDLDADTMAKQIREWAQAG
MTR                TLSGQTGEGFVISVSHGEPLCIGLNCALGAAEMR--PFIEIIGKC-TTAYVLCYPNAGLPNTFGDYDETPSMMAKHLKDFAMDG

cons                                                        : :        .....    :* ::.  .. : *


PF3D7_1233700      ---------------------GEKKKMKKNKEKKEEKKNKYINDNLCNFVDEK-------------------------------
PKH_145080         -------DG----GGVVQPDKAVVGCR---------------------------------------------------------
PmUG01_14067900    -------KR----ENLAIHGKVITGNK------------------LASLKYDET------------------------------
PowCR01_1400537    --------------------PSERKKK---------------------------------------------------------
PocGH01_1405930    --------------------PSERKKK---------------------------------------------------------
PVX_100640         ---------------AAPLGNAMTSCK---------------------------------------------------------
metH               FLNIVGGCCGTTPQHIAAMSRAVEGLA------------------PRKLPEIPVA--CRLSGLEPLNIGEDSLFVNVGERTNVT
MTR                LVNIVGGCCGSTPDHIREIAEAVKNCK------------------PRVPPATAFEGHMLLSGLEPFRIGPYTNFVNIGERCNVA

cons


PF3D7_1233700      --------------------SQGKKKKNMSEYYQLDKNIIKNKNNIDRNIKKSINYKAYYKYSLYYDIQMNISSVNSYGREKYN
PKH_145080         ------------------------------------------------------------SQNQSYDIIIKTPP----------
PmUG01_14067900    --------------------GKNKTDDFT---------------------------KMLNKQIKHFDIKLNILSE---------
PowCR01_1400537    ------------------------------------------------------------RNVHTYDIEVNIPSINKHGVG---
PocGH01_1405930    ------------------------------------------------------------RNVHIYDIEVNIPCKNKHDIE---
PVX_100640         ------------------------------------------------------------PQNQQYDIFISTPT----------
metH               GSAKFKRLIKEEKYSEALDVARQQVE----------------------------------NGAQIIDINMDEGMLDAE------
MTR                GSRKFAKLIMAGNYEEALCVAKVQVE----------------------------------MGAQVLDVNMDDGMLDGP------

cons                                                                 *: :.
```

```
PF3D7_1233700        PIEIVPKMKKKIQ--NVNCTNKDETNIINTHN-----------N--------------NNNDNIFFNKKLFNYGIEYYIDVSDQ
PKH_145080           ------EVEGSLMHRQNQLPDIEMVPL-SSTQ----------RS--------------ATSTEDFPNKHFFNYGLEYYIDVRDE
PmUG01_14067900      --------YTTRN--MTKRSSVEFIPI-ARRY-----------D--------------KKRCNMVASPKLFNYGLEYYIDVSDE
PowCR01_1400537      --AAIPRCKVHLQ--HSNRSMIQMTPR-SGMK-----------S--------------KEKCNWLRNWKLFNYGIEYYIDVSDM
PocGH01_1405930      --ANIPRCKVHLQ--HSSRSIIQMTPR-SGMK-----------S--------------KEKCNWLLNWKLFNYGIEYYIDVSDM
PVX_100640           ------------LHADDQPPSIKMIPV-SSRESGRE------RG--------------GPSREAAPNRHLFNYGLEYYVDVGDE
metH                 ------AAMVRFLNLIAGEPDIARVPI-MIDSSKWDVIEKGLKCIQGKGIVNSISMKEGVDAFIHHAKLLRRYGAAVVVMAFDE
MTR                  ------SAMTRFCNLIASEPDIAKVPL-CIDSSNFAVIEAGLKCCQGKCIVNSISLKEGEDDFLEKARKIKKYGAAMVVMAFDE

cons                                    .                                                 : .**    : . *

PF3D7_1233700        EI------------------------------------------------------------------------ISNCLFKLNT
PKH_145080           EI------------------------------------------------------------------------ISNSKFRIDS
PmUG01_14067900      EI------------------------------------------------------------------------ASNCKFKLQC
PowCR01_1400537      EV------------------------------------------------------------------------ISNCKLKLEC
PocGH01_1405930      EV------------------------------------------------------------------------ISNCKFKLEC
PVX_100640           EI------------------------------------------------------------------------ISNCNFKLGA
metH                 QGQADTRARKIEICRRAYKILTEEVGFPPEDIIFDPNIFAVATGIEEHNNYAQDFIGACEDIKRELPHALISGGVSNVSFSFRG
MTR                  EGQATETDTKIRVCTRAYHLLVKKLGFNPNDIIFDPNILTIGTGMEEHNLYAINFIHATKVIKETLPGARISGGLSNLSFSFRG

cons                 :                                                                          **  : :

PF3D7_1233700        FCKNSDKLH---------------------------------------------------------------------------
PKH_145080           YKRNEHKLQ---------------------------------------------------------------------------
PmUG01_14067900      FCRNTNKLN---------------------------------------------------------------------------
PowCR01_1400537      FTRNREKLH---------------------------------------------------------------------------
PocGH01_1405930      FTRNREKLH---------------------------------------------------------------------------
PVX_100640           YKRNEEKLH---------------------------------------------------------------------------
metH                 NDPVREAIHAVFLYYAIRNGMDMGIVNAGQLAIYDDLPAELRDAVEDVILNRRDDGTERLLELAEKYRGSKTDDTANAQQAEWR
MTR                  MEAIREAMHGVFLYHAIKSGMDMGIVNAGNLPVYDDIHKELLQLCEDLIWNKDPEATEKLLRYAQT-QG--TGGKKVIQTDEWR

cons                      . ::

PF3D7_1233700        --------------------------------------------------------------LFSLTTFSNIRE-----VLTFY
PKH_145080           --------------------------------------------------------------LFSVITSSNVRE-----VFTLY
PmUG01_14067900      --------------------------------------------------------------FFSLTTFSNIRE-----VLTFY
PowCR01_1400537      --------------------------------------------------------------FFSLTTSSNVRE-----IFTFY
PocGH01_1405930      --------------------------------------------------------------FFSLTTSSNVRE-----IFTFY
PVX_100640           --------------------------------------------------------------LFSLLTSSNVRE-----VLTLY
metH                 SWEVNKRLEYSLVKGITEFIEQDTEEAR---QQATRPIEVIEGPLMDGMNVVGDLFGEGKMFLPQVVKSARVMKQAVAYLEPFI
MTR                  NGPVEERLEYALVKGIEKHIIEDTEEARLNQKKYPRPLNIIEGPLMNGMKIVGDLFGAGKMFLPQVIKSARVMKKAVGHLIPFM

cons                                                                                 : .: . :.: :     : .:

PF3D7_1233700        NC----------IKYYGSTFNNKVIINFFCNSCKYVGCSKYSFFDIVSILLYLDSYNKYIKAIGLNCVNIEYV-YDLFYPFQKY
PKH_145080           HH----------LKTCGGGFEKNVVVSFYCNSNKNIGCTDYSFLDMVLTLLYLDSRNHFIKAIGVNCVNIDYV-RELILPLTRC
PmUG01_14067900      NY----------IKYYASNFKNNVIINFYCNSSKFIGCSDYSFFDIVSILLYLDSYNKFINAIGINCVNIENV-YDLFFPFKKY
PowCR01_1400537      NF----------LKYYGNNFRNSVIINIFCNDIYHIGCSIYSFFDIICILLYLDAHNKYIYAIGLNCVNIEYV-YDIFLPFKKY
PocGH01_1405930      NF----------LKYYGNNFRNSVIINIFCNDIYHIGCSIYSFFDIICILLYLDAHNKYIYAIGLNCVNIEYV-YDIFLPFKKY
PVX_100640           SH----------LVKCGGHFDTNVVVSFYCNDGQHIGCSGYSFVDVVLILLYLDSRNRFIKAIGLNCVSIDSV-RELFAPLTRC
metH                 EASKE-----------QGKTNGKMVIATVKGDVHDIG--------------------KNIVGVVLQCNNYEIVDLGVMVPAEKI
MTR                  EKEREETRVLNGTVEEEDPYQGTIVLATVKGDVHDIG--------------------KNIVGVVLGCNNFRVIDLGVMTPCDKI

cons                                  .    .:::    ..   :*                    : * .: : * .   :   :: *  :

PF3D7_1233700        ICPYNNINT---------------------------------------------------------------------------
PKH_145080           IKPDGNIDV---------------------------------------------------------------------------
PmUG01_14067900      TCPYININE---------------------------------------------------------------------------
PowCR01_1400537      LCPYKNINV---------------------------------------------------------------------------
PocGH01_1405930      LCPYKNIIV---------------------------------------------------------------------------
PVX_100640           ISSDGTVDV---------------------------------------------------------------------------
metH                 LRTAKEVNADLIGLSGLITPSLDEMVNVAKEMERQGFTIPLLIGGATTSKAHTAVKIEQNYSGPTVYVQNASRTVGVVAALLSD
MTR                  LKAALDHKADIIGLSGLITPSLDEMIFVAKEMERLAIRIPLLIGGATTSKTHTAVKIAPRYSAPVIHVLDASKSVVVCSQLLDE

cons                   .

PF3D7_1233700        ------------------------------------------------------------------------------------
PKH_145080           ------------------------------------------------------------------------------------
PmUG01_14067900      ------------------------------------------------------------------------------------
PowCR01_1400537      ------------------------------------------------------------------------------------
PocGH01_1405930      ------------------------------------------------------------------------------------
PVX_100640           ------------------------------------------------------------------------------------
metH                 TQRDDFVARTRKEYETVRIQHGRKKPRTPPVTLEAARDNDFAFDWQAYTPPVAH-RLGVQEVEA-SIETLRNYIDWTPFFMTWS
MTR                  NLKDEYFEEIMEEYEDIRQDHYESLKERRYLPLSQARKSGFQMDWLSEPHPVKPTFIGTQVFEDYDLQKLVDYIDWKPFFDVWQ

cons

PF3D7_1233700        ------------------------------------------------------------------------------------
PKH_145080           ------------------------------------------------------------------------------------
PmUG01_14067900      ------------------------------------------------------------------------------------
PowCR01_1400537      ------------------------------------------------------------------------------------
PocGH01_1405930      ------------------------------------------------------------------------------------
```

```
PVX_100640         ------------------------------------------------------------------------------------
metH               LAGKYP-----RILEDEVVGVEAQRLFKDANDMLDKLSAEKTLNPRGVVGLFPANRVGDDIEIYRDET---RTHVINVSHHLRQ
MTR                LRGKYPNRGFPKIFNDKTVGGEARKVYDDAHNMLNTLISQKKLRARGVVGFWPAQSIQDDIHLYAEAAVPQAAEPIATFYGLRQ

cons

PF3D7_1233700      ---------------------------------------------NLYHSQNIQMNNIVKDVMKDLKKNRYIND-------FNF
PKH_145080         ---------------------------------------------NAYPCPNGELGRLIKTVLKDLKKNTYLGD-------IHF
PmUG01_14067900    ---------------------------------------------DSYKSQNSQINSIVKTALRSLKKNRYIQD-------VNF
PowCR01_1400537    ---------------------------------------------NMYRSENQEFDSIMKTVLNDLNKNRYIYD-------VNL
PocGH01_1405930    ---------------------------------------------NMYRSENQEFTSIMKTVLNDLNKNRYIYD-------VNF
PVX_100640         ---------------------------------------------DAYGSPSGELSGLTKTILKGLKKNRFLGD-------IHF
metH               QTEKTGFAN---YCLADFVAPKLSGKADYIGAFAVTGGLEEDALADAFEAQHDDYNKIMVKALADRLAEAFAEYLHERVRKVYW
MTR                QAEKDSASTEPYYCLSDFIAPLHSGIRDYLGLFAVACF-GVEELSKAYEDDGDDYSSIMVKALGDRLAEAFAEELHERVRRELW

cons                                                            . :     :   :    : .   : :

PF3D7_1233700      F---------------------------------------------------------CSPNKSLEVVSFDDQNGDVHF-----
PKH_145080         F---------------------------------------------------------ASPNKSLNRVTYDHSRNDIQF-----
PmUG01_14067900    F---------------------------------------------------------CSPNKSLQRVSFDNSTNEVTF-----
PowCR01_1400537    F---------------------------------------------------------CSPNKSLQRVTYDYNTNHVNF-----
PocGH01_1405930    F---------------------------------------------------------CSPNKSLQRVTYDYNTNHVNF-----
PVX_100640         F---------------------------------------------------------ASPNKSLNRVTYDYQGGEVHF-----
metH               GYAPNENLSNEELIRENYQGIRPAPGYPACPEHTEKATIWELLEVEKHTGMKLTESFAMWPGASVSGWYFSHPD-SKYYAVAQI
MTR                AYCGSEQLDVADLRRLRYKGIRPAPGYPSQPDHTEKLTMWRLADIEQSTGIRLTESLAMAPASAVSGLYFSNLK-SKYFAVGKI

cons                                                                           *  ::.   :.       :

PF3D7_1233700      -----HNSANKTNHVYNYIEKWINVHVNGFGGCCYYNPYDISLINYKINELL----------
PKH_145080         -----DISQKRHKHLYNYVGDWIDVGLTGFGGCCYYNPYDISLLDYKLGRMAQMQ-------
PmUG01_14067900    -----HTLPNKTDHVYNYVDKWMEVGINGFGGCCYYNPYDVSLIDYKLGRLCR---------
PowCR01_1400537    -----ETTSKKINHVYNYLERWIDLGINGFGGCCYYNPYDISLIDYKLSSIFSGGTN-----
PocGH01_1405930    -----ETTSKRINHVYNYLESWIDLGINGFGGCCYYNPYDISLIDYKLSSIFSSGTN-----
PVX_100640         -----ETTQERHKHVCNYVGEWLHVGLSGFGGCCYYNPYDISLLDYKLGQLAGGVAVREKQR
metH               QRDQVEDYARRKGMSVTEVERWLAPNL-G---------YDAD--------------------
MTR                SKDQVEDYALRKNISVAEVEKWLGPIL-G---------YDTD--------------------

cons                    .    :       :  *:   : *         ** .
```

Fig. S1.
