# Supplementary figures and images for: Antimalarial target vulnerability of the putative Plasmodium falciparum methionine synthase
Source: PeerJ. 2024 Jan 15;12:e16595. doi: 10.7717/peerj.16595 (PMC10795524; doi:10.7717/peerj.16595)

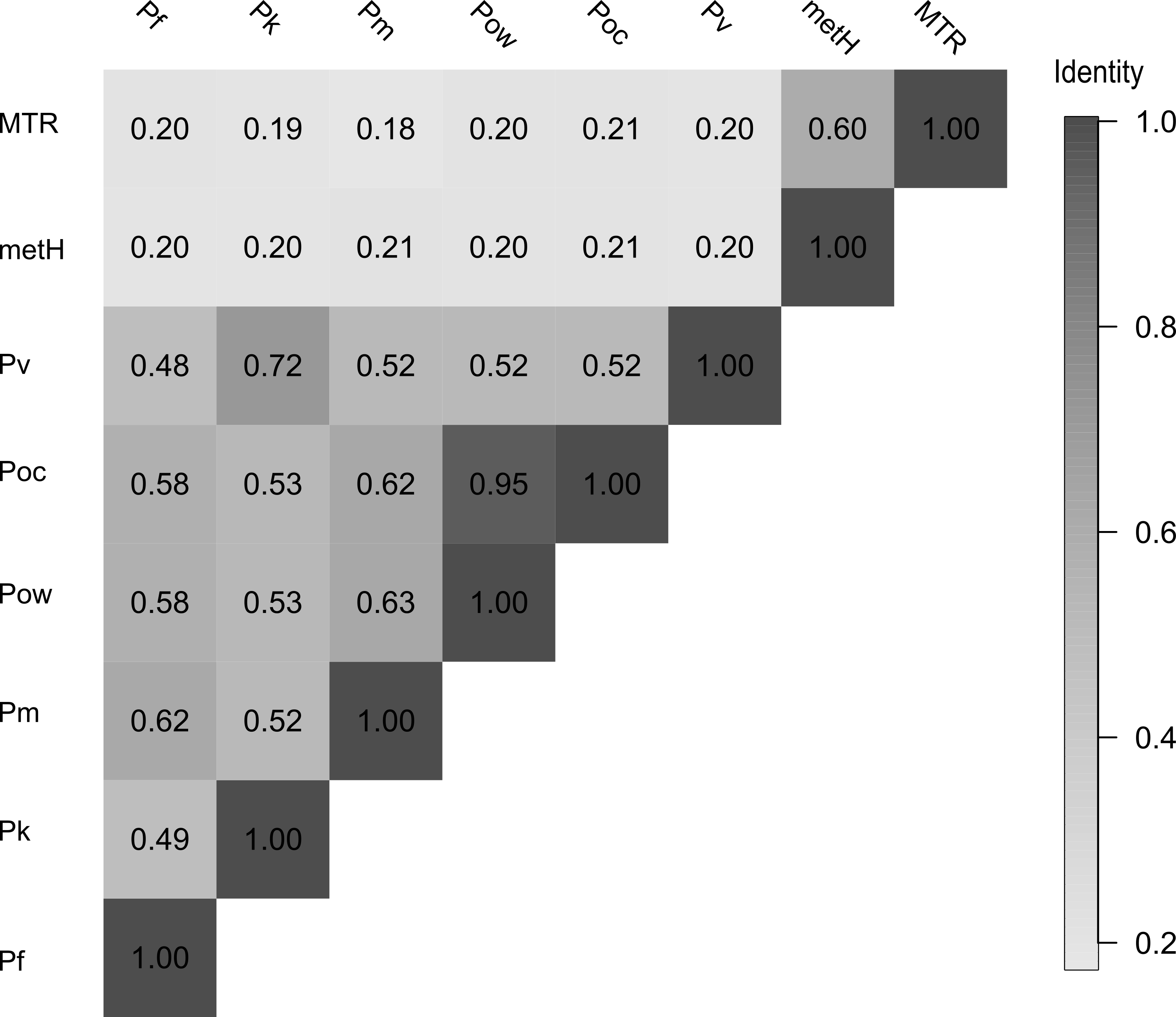

Supplement: Supplemental Information 2 — Percent identity matrix of the protein alignment (Fig. S1) was created using the bio3d R package version 2.4-4. Cells are shaded according to the percent identity as indicated by the scale bar on the right. [file peerj-12-16595-s002.png]

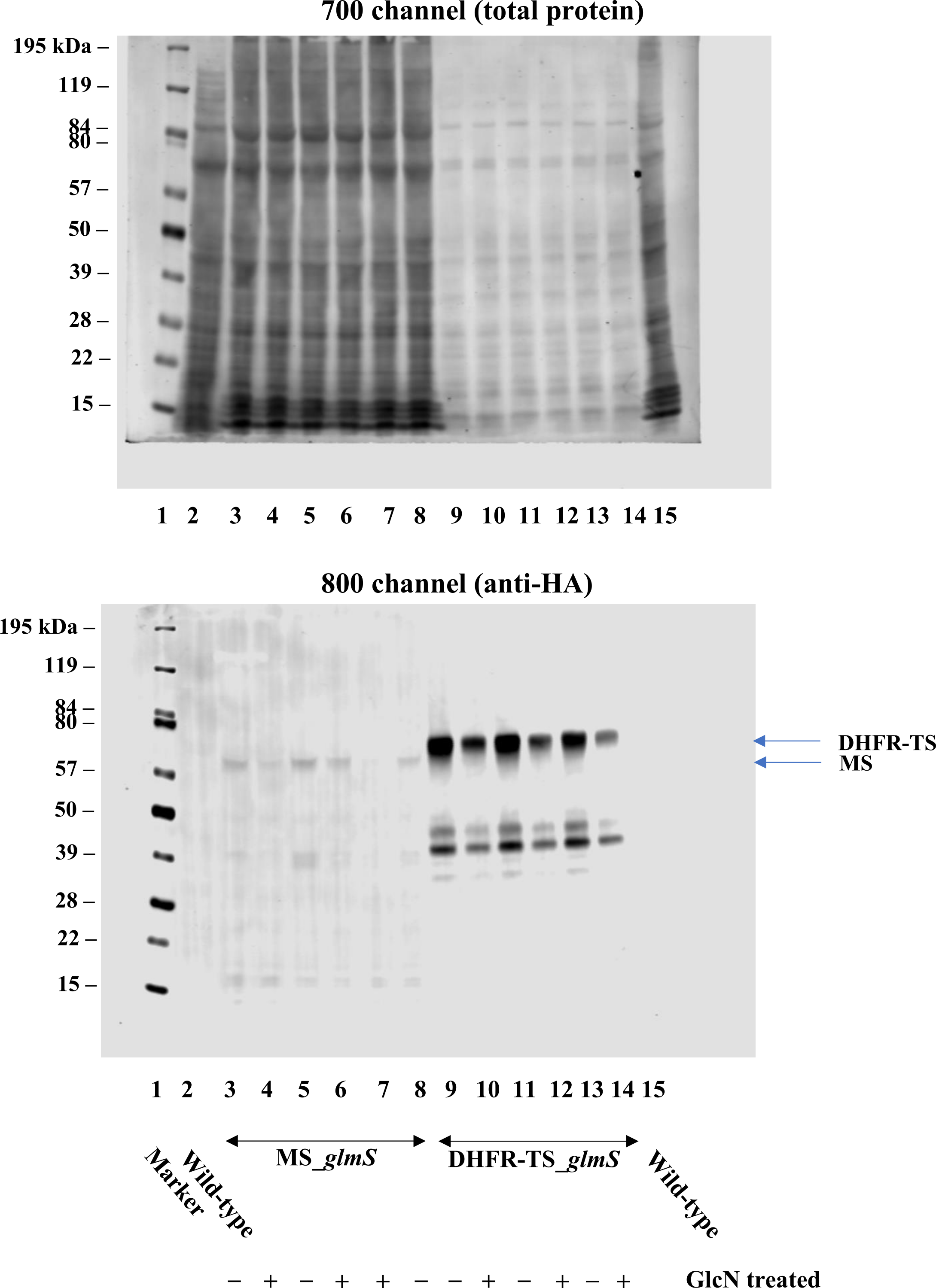

Supplement: Supplemental Information 3 — Clonal lines of transgenic parasites DHFR-TS_glmS and MS_glmS with edited PF3D7_0417200 (DHFR-TS) and PF3D7_1233700 (MS) genes, respectively were cultured for 24 h in the presence or absence of 5 mM glucosamine (GlcN). A sample of parasite protein extract (50, 50, and 2.5 µg of total protein from 3D7 wild-type, MS_glmS and DHFR-TS_glmS transgenic parasites, respectively) was separated in each lane of a 4–12% NuPAGE Bis-Tris protein gel in MOPS running buffer (Invitrogen). Upper panel shows total protein staining with REVERT (700 channel). Lower panel shows target protein signal of the same membrane detected with anti-HA antibody (800 channel). The images are uncropped and unedited. Total protein and target protein band (HA-tagged MS = 72.7 kDa and HA-tagged DHFR-TS = 75.5 kDa) intensities were determined using Image Studio v5.2 (LI-COR Biosciences). Migrations of DHFR-TS and MS target protein bands are indicated on the right. Lane designations:- Lane 1: iBright™ Prestained Protein Ladder (Invitrogen, Thermo Fischer Scientific) Lane 2: 3D7 wild-type Lane 3: MS_glmS parasite (-) GlcN (replicate #1) Lane 4: MS_glmS parasite (+) GlcN (replicate #1) Lane 5: MS_glmS parasite (-) GlcN (replicate #2) Lane 6: MS_glmS parasite (+) GlcN (replicate #2) Lane 7: MS_glmS parasite (+) GlcN (replicate #3) Lane 8: MS_glmS parasite (-) GlcN (replicate #3) Lane 9: DHFR-TS_glmS parasite (-) GlcN (replicate #1) Lane 10: DHFR-TS_glmS parasite (+) GlcN (replicate #1) Lane 11: DHFR-TS_glmS parasite (-) GlcN (replicate #2) Lane 12: DHFR-TS_glmS parasite (+) GlcN (replicate #2) Lane 13: DHFR-TS_glmS parasite (-) GlcN (replicate #3) Lane 14: DHFR-TS_glmS parasite (+) GlcN (replicate #3) Lane 15: 3D7 wild-type [file peerj-12-16595-s003.png]
