# Supplementary material for: Antimalarial target vulnerability of the putative Plasmodium falciparum methionine synthase
Source: PeerJ. 2024 Jan 15;12:e16595. doi: 10.7717/peerj.16595 (PMC10795524; doi:10.7717/peerj.16595)
Supplement: Supplementary file 3 [file peerj-12-16595-s006.odt]

>px3HA_glmS (5909 bp),

ggtaccTACCCGTACGACGTCCCGGACTACGCTGGCTATCCCTATGATGTGCCCGATTATGCGTATCCGTACGATGTTCCAGATTATGCCGCTGCTAGCTAACCATGGCGCGACGCGTACTAGCTAACCATGGCGCGACGCGTACTAGTCACATATAATTATAGCGCCCGAACTAAGCGCCCGGAAAAAGGCTTAGTTGACGAGGATGGAGGTTATCGAATTTTCGGCGGATGCCTCCCGGCTGAGTGTGCAGATCACAGCCGTAAGGATTTCTTCAAACCAAGGGGGTGACTCCTTGAACAAAGAGAAATCACATGATCTTCCAAAAAACATGTAGGAAGGGGACTCGAGGGATATGGCAGCTTAATGTTCGTTTTTCTTATTTATATATTTATACCAATTGATTGTATTTATAACTGTAAAAATGTGTATGTTGTGTGCATATTTTTTTTTGTGCATGCACATGCATGTAAATAGCTAAAATTATGAACATTTTATTTTTTGTTCAGAAAAAAAAAACTTTACACACATAAAATGGCTAGTATGAATAGCCATATTTTATATAAATTAAATCCTATGAATTTATGACCATATTAAAAATTTAGATATTTATGGAACATAATATGTTTGAAACAATAAGACAAAATTATTATTATTATTATTATTTTTACTGTTATAATTATGTGTCTCCTTCAATGATTCATAAATAGTTGGACTTGATTTTTAAAATGTTTATAATATGATTAGCATAGTTAAATAAAAAAAGTTGAAAAATTAAAAAAAAACATATAAACACAAATGATGGTTTTTCCTTCAATTTCGATATCATATAGCAATCGTATTAGCAGGTAAATGAGCAAGGTTAATAAAAAAATGGTAAAAAAAAAAAATTGGGGAAAAATAAACAAGGTGTCTTTATCTACACTTTGGCTTTACAATGTATAATTTATATGCATACAAAAAAATTTTGATAAAATTGTAAAAAGGTGAATAAAATAACATTGTAACAGTAGTAGATAGTAAAGGGAAGGTGTTGCTCAAATAGTGTCGAAACAAAACTGGCATAAAGAAAAATTGATATTGAGCAGAGGATATGCGCATAATGGTATTTTGTTTGTTTGTTTGTTTTCTCATTTTTTGAGACAGCTCAATTCTTTATGTCCACAACATCATCGGACTTTTCTTCTTCAGGGTAGGCGGCCGCAGCTTGAGTATTCTATAGTGTCACCTAAATAGCTTGGCGTAATCATGGTCATAGCTGTTTCCTGTGTGAAATTGTTATCCGCTCACAATTCCACACAACATACGAGCCGGAAGCATAAAGTGTAAAGCCTGGGGTGCCTAATGAGTGAGCTAACTCACATTAATTGCGTTGCGCTCACTGCCCGCTTTCCAGTCGGGAAACCTGTCGTGCCAGCTGCATTAATGAATCGGCCAACGCGCGGGGAGAGGCGGTTTGCGTATTGGGCGCTCTTCCGCTTCCTCGCTCACTGACTCGCTGCGCTCGGTCGTTCGGCTGCGGCGAGCGGTATCAGCTCACTCAAAGGCGGTAATACGGTTATCCACAGAATCAGGGGATAACGCAGGAAAGAACATGTGAGCAAAAGGCCAGCAAAAGGCCAGGAACCGTAAAAAGGCCGCGTTGCTGGCGTTTTTCGATAGGCTCCGCCCCCCTGACGAGCATCACAAAAATCGACGCTCAAGTCAGAGGTGGCGAAACCCGACAGGACTATAAAGATACCAGGCGTTTCCCCCTGGAAGCTCCCTCGTGCGCTCTCCTGTTCCGACCCTGCCGCTTACCGGATACCTGTCCGCCTTTCTCCCTTCGGGAAGCGTGGCGCTTTCTCATAGCTCACGCTGTAGGTATCTCAGTTCGGTGTAGGTCGTTCGCTCCAAGCTGGGCTGTGTGCACGAACCCCCCGTTCAGCCCGACCGCTGCGCCTTATCCGGTAACTATCGTCTTGAGTCCAACCCGGTAAGACACGACTTATCGCCACTGGCAGCAGCCACTGGTAACAGGATTAGCAGAGCGAGGTATGTAGGCGGTGCTACAGAGTTCTTGAAGTGGTGGCCTAACTACGGCTACACTAGAAGGACAGTATTTGGTATCTGCGCTCTGCTGAAGCCAGTTACCTTCGGAAAAAGAGTTGGTAGCTCTTGATCCGGCAAACAAACCACCGCTGGTAGCGGTGGTTTTTTTGTTTGCAAGCAGCAGATTACGCGCAGAAAAAAAGGATCTCAAGAAGATCCTTTGATCTTTTCTACGGGGTCTGACGCTCAGTGGAACGAAAACTCACGTTAAGGGATTTTGGTCATGAGATTATCAAAAAGGATCTTCACCTAGATCCTTTTAAATTAAAAATGAAGTTTTAAATCAATCTAAAGTATATATGAGTAAACTTGGTCTGACAGTTACCAATGCTTAATCAGTGAGGCACCTATCTCAGCGATCTGTCTATTTCGTTCATCCATAGTTGCCTGACTCCCCGTCGTGTAGATAACTACGATACGGGAGGGCTTACCATCTGGCCCCAGTGCTGCAATGATACCGCGAGACCCACGCTCACCGGCTCCAGATTTATCAGCAATAAACCAGCCAGCCGGAAGGGCCGAGCGCAGAAGTGGTCCTGCAACTTTATCCGCCTCCATCCAGTCTATTAATTGTTGCCGGGAAGCTAGAGTAAGTAGTTCGCCAGTTAATAGTTTGCGCAACGTTGTTGGCATTGCTACAGGCATCGTGGTGTCACGCTCGTCGTTTGGTATGGCTTCATTCAGCTCCGGTTCCCAACGATCAAGGCGAGTTACATGATCCCCCATGTTGTGCAAAAAAGCGGTTAGCTCCTTCGGTCCTCCGATCGTTGTCAGAAGTAAGTTGGCCGCAGTGTTATCACTCATGGTTATGGCAGCACTGCATAATTCTCTTACTGTCATGCCATCCGTAAGATGCTTTTCTGTGACTGGTGAGTACTCAACCAAGTCATTCTGAGAATAGTGTATGCGGCGACCGAGTTGCTCTTGCCCGGCGTCAATACGGGATAATACCGCGCCACATAGCAGAACTTTAAAAGTGCTCATCATTGGAAAACGTTCTTCGGGGCGAAAACTCTCAAGGATCTTACCGCTGTTGAGATCCAGTTCGATGTAACCCACTCGTGCACCCAACTGATCTTCAGCATCTTTTACTTTCACCAGCGTTTCTGGGTGAGCAAAAACAGGAAGGCAAAATGCCGCAAAAAAGGGAATAAGGGCGACACGGAAATGTTGAATACTCATACTCTTCCTTTTTCAATATTATTGAAGCATTTATCAGGGTTATTGTCTCATGAGCGGATACATATTTGAATGTATTTAGAAAAATAAACAAATAGGGGTTCCGCGCACATTTCCCCGAAAAGTGCCACCTGACGTCTAAGAAACCATTATTATCATGACATTAACCTATAAAAATAGGCGTATCACGAGGCCCTTTCGTCTCGCGCGTTTCGGTGATGACGGTGAAAACCTCTGACACATGCAGCTCCCGGAGACGGTCACAGCTTGTCTGTAAGCGGATGCCGGGAGCAGACAAGCCCGTCAGGGCGCGTCAGCGGGTGTTGGCGGGTGTCGGGGCTGGCTTAACTATGCGGCATCAGAGCAGATTGTACTGAGAGTGCACCATATGCGGTGTGAAATACCGCACAGATGCGTAAGGAGAAAATACCGCATCAGGCGCCATTCGCCATTCAGGCTGCGCAACTGTTGGGAAGGGCGATCGGTGCGGGCCTCTTCGCTATTACGCCAGCTGGCGAAAGGGGGATGTGCTGCAAGGCGATTAAGTTGGGTAACGCCAGGGTTTTCCCAGTCACGACGTTGTAAAACGACGGCCAGTGAATTGTAATACGACTCACTATAGAATTCTAGATTTAATAAATATGTTCTTATATATAATGAGAAATAAATATTTAACATATGTTTAAAAAGAAAAATTTAAGATTTACATGATTAGGAATAAAAATATTAAAAGCTATATATTAATGTTGTACTTTATGTTCATATTGTAAAAGATAAATATAATTTTTATTAAAATTTATATTTATTATTATTATAAGTTCTTAGAAAATACATTTTGTTTTAAAATAATACTATAAAAAGAACAATAATAAAAATAAAATACAATTAAAACATAATTAAAAAAGAAAAATACAGACAGTAAAAAAAATCGCTATCCCATAAATTACAAAACATGAATTAATAAACATATATTGTTTATTAATAAAATTGTAAGTTATTTTTATATATACATAATAAATTAAAGATACTGAAACAATAATTTTAAATGTAAATAAATGAATAATGATATGTTTTATATGATTCATTATTCTATATTTATAAGGAAGATTACAAAAAAAAAATTCATATGTATTTTTTTTGTAATTTCTGTGTTTATGTTTTTATTTTTATAATATTTTTAATCTATTATTAAATAAGCTTTTAGCCCTCCCACACATAACCAGAGGGCAGCAATTCACGAATCCCAACTGCCGTCGGCTGTCCATCACTGTCCTTCACTATCGCTTTGATCCCAGGATGCAGATCGAGAAGCACCTGTCGACACCGTCCGCAGGGGCTCAAGATGCCCCTGTTCTCATTTCCGATCGCGACGATACAAGTCAGGTTGCCAGCTGCCGCAGCAGCAGCAGTGCCCAGCACCACGAGTTCTGCACAAGGTCCCCCAGTAAAATGATATACATTGACACCAGTGAAGATGCGGCCGTCGCTAGAGAGAGCTGCGCTGGCGACGCTGTAGTCTTCAGAGATGGGGATGCTGTTGATTGTAGCCGTTGCTCTTTCAATGAGGGTGGATTCTTCTTGAGACAAAGGCATGGATCCTGATATATTTCTATTAGGTATTTATTATTATAAAATATAAATCTTGAATGATAATAAATAAAATATTAGTTATTCCTTTTCTAGTTTAAAATATACATATTATAAATATATATATATATATATATATTTTTATTGTGACAAGAATATATAATTATAAATTATATTATTTATTTTTGTATTTTTTTTTTTTTTTTTTTTTTTTTCTTTTTTTGTTTTATTTTTCTTTTTTTTTATAAATATTATTTTTTTCTTTTATCATGCACATTGGAATAATACATTAATATATATATATATATTATATTATACATATATTGAATAATGTTTATAAAAAATGCATAACTTATATGAATATAATTTTTTTTAAATATGACAAAAAGAAAAAAAAAAAAAACCAAAAAAAATTAAAATTGAAATGAAATATATAAATATATTATTTATATATATTATACATTGTTTAATACTACTACATGTATATATATATATTATATATATATATATATATCAATTTTTTCAAAAATAAATTAATATAAAAAGAGGGGAAAAAAAAAAAAAAAAAAAAAAAAAGATAATTAAGTAAGCATTTAAAAATATATAAATTGATAATATATAAAATTAATCACATATAAACTAATATAATTTATAAAATAAGGAAAATAAAATATTACCATAAAATAAAAATAAAAATAAAAAAAAAAAAAAAAAACACCTTTTTTTATATATATTAATATATAATTATCTCTTAGAAAAAATATTGTATAATTATATATGTAATGATTTATATAAAAAAATAAAATTATACAAGTATATATTTTGTTTCTATAAATTGATACATAACTTAACTAAGGTCTTACTTTTACTAACTTAGGTCATACTTCTACTAACATAAGTCTTAACTTACCTAACATAGGTCTTAACTTGACTAATCTAGGTATGAACTTCAATAACTAAGGTCTTAACATGACTAACATAGGTCTTAACTTAACTAACTTAGGTCATACTTCGATTAACTAAGGTCTTACATTCACTGATATAGGTCTTAA
