# Supplementary material for: Antimalarial target vulnerability of the putative Plasmodium falciparum methionine synthase
Source: PeerJ. 2024 Jan 15;12:e16595. doi: 10.7717/peerj.16595 (PMC10795524; doi:10.7717/peerj.16595)
Supplement: Supplementary file 5 [file peerj-12-16595-s008.odt]

>MS_repair_vector (7429 bp),

gtacctacccgtacgacgtcccggactacgctggctatccctatgatgtgcccgattatgcgtatccgtacgatgttccagattatgccgctgctagctaaccatggcgcgacgcgtactagctaaccatggcgcgacgcgtactagtcacatataattatagcgcccgaactaagcgcccggaaaaaggcttagttgacgaggatggaggttatcgaattttcggcggatgcctcccggctgagtgtgcagatcacagccgtaaggatttcttcaaaccaagggggtgactccttgaacaaagagaaatcacatgatcttccaaaaaacatgtaggaaggggactcgagggatatggcagcttaatgttcgtttttcttatttatatatttataccaattgattgtatttataactgtaaaaatgtgtatgttgtgtgcatatttttttttgtgcatgcacatgcatgtaaatagctaaaattatgaacattttattttttgttcagaaaaaaaaaactttacacacataaaatggctagtatgaatagccatattttatataaattaaatcctatgaatttatgaccatattaaaaatttagatatttatggaacataatatgtttgaaacaataagacaaaattattattattattattatttttactgttataattatgtgtctccttcaatgattcataaatagttggacttgatttttaaaatgtttataatatgattagcatagttaaataaaaaaagttgaaaaattaaaaaaaaacatataaacacaaatgatggtttttccttcaatttcgatatcatatagcaatcgtattagcaggtaaatgagcaaggttaataaaaaaatggtaaaaaaaaaaaattggggaaaaataaacaaggtgtctttatctacactttggctttacaatgtataatttatatgcatacaaaaaaattttgataaaattgtaaaaaggtgaataaaataacattgtaacagtagtagatagtaaagggaaggtgttgctcaaatagtgtcgaaacaaaactggcataaagaaaaattgatattgagcagaggatatgcgcataatggtattttgtttgtttgtttgttttctcattttttgagacagctcaattctttatgtccacaacatcatcggacttttcttcttcagggtaggcggccgcagcttgagtattctatagtgtcacctaaatagcttggcgtaatcatggtcatagctgtttcctgtgtgaaattgttatccgctcacaattccacacaacatacgagccggaagcataaagtgtaaagcctggggtgcctaatgagtgagctaactcacattaattgcgttgcgctcactgcccgctttccagtcgggaaacctgtcgtgccagctgcattaatgaatcggccaacgcgcggggagaggcggtttgcgtattgggcgctcttccgcttcctcgctcactgactcgctgcgctcggtcgttcggctgcggcgagcggtatcagctcactcaaaggcggtaatacggttatccacagaatcaggggataacgcaggaaagaacatgtgagcaaaaggccagcaaaaggccaggaaccgtaaaaaggccgcgttgctggcgtttttcgataggctccgcccccctgacgagcatcacaaaaatcgacgctcaagtcagaggtggcgaaacccgacaggactataaagataccaggcgtttccccctggaagctccctcgtgcgctctcctgttccgaccctgccgcttaccggatacctgtccgcctttctcccttcgggaagcgtggcgctttctcatagctcacgctgtaggtatctcagttcggtgtaggtcgttcgctccaagctgggctgtgtgcacgaaccccccgttcagcccgaccgctgcgccttatccggtaactatcgtcttgagtccaacccggtaagacacgacttatcgccactggcagcagccactggtaacaggattagcagagcgaggtatgtaggcggtgctacagagttcttgaagtggtggcctaactacggctacactagaaggacagtatttggtatctgcgctctgctgaagccagttaccttcggaaaaagagttggtagctcttgatccggcaaacaaaccaccgctggtagcggtggtttttttgtttgcaagcagcagattacgcgcagaaaaaaaggatctcaagaagatcctttgatcttttctacggggtctgacgctcagtggaacgaaaactcacgttaagggattttggtcatgagattatcaaaaaggatcttcacctagatccttttaaattaaaaatgaagttttaaatcaatctaaagtatatatgagtaaacttggtctgacagttaccaatgcttaatcagtgaggcacctatctcagcgatctgtctatttcgttcatccatagttgcctgactccccgtcgtgtagataactacgatacgggagggcttaccatctggccccagtgctgcaatgataccgcgagacccacgctcaccggctccagatttatcagcaataaaccagccagccggaagggccgagcgcagaagtggtcctgcaactttatccgcctccatccagtctattaattgttgccgggaagctagagtaagtagttcgccagttaatagtttgcgcaacgttgttggcattgctacaggcatcgtggtgtcacgctcgtcgtttggtatggcttcattcagctccggttcccaacgatcaaggcgagttacatgatcccccatgttgtgcaaaaaagcggttagctccttcggtcctccgatcgttgtcagaagtaagttggccgcagtgttatcactcatggttatggcagcactgcataattctcttactgtcatgccatccgtaagatgcttttctgtgactggtgagtactcaaccaagtcattctgagaatagtgtatgcggcgaccgagttgctcttgcccggcgtcaatacgggataataccgcgccacatagcagaactttaaaagtgctcatcattggaaaacgttcttcggggcgaaaactctcaaggatcttaccgctgttgagatccagttcgatgtaacccactcgtgcacccaactgatcttcagcatcttttactttcaccagcgtttctgggtgagcaaaaacaggaaggcaaaatgccgcaaaaaagggaataagggcgacacggaaatgttgaatactcatactcttcctttttcaatattattgaagcatttatcagggttattgtctcatgagcggatacatatttgaatgtatttagaaaaataaacaaataggggttccgcgcacatttccccgaaaagtgccacctgacgtctaagaaaccattattatcatgacattaacctataaaaataggcgtatcacgaggccctttcgtctcgcgcgtttcggtgatgacggtgaaaacctctgacacatgcagctcccggagacggtcacagcttgtctgtaagcggatgccgggagcagacaagcccgtcagggcgcgtcagcgggtgttggcgggtgtcggggctggcttaactatgcggcatcagagcagattgtactgagagtgcaccatatgcggtgtgaaataccgcacagatgcgtaaggagaaaataccgcatcaggcgccattcgccattcaggctgcgcaactgttgggaagggcgatcggtgcgggcctcttcgctattacgccagctggcgaaagggggatgtgctgcaaggcgattaagttgggtaacgccagggttttcccagtcacgacgttgtaaaacgacggccagtgaattgtaatacgactcactatagaattctagatttaataaatatgttcttatatataatgagaaataaatatttaacatatgtttaaaaagaaaaatttaagatttacatgattaggaataaaaatattaaaagctatatattaatgttgtactttatgttcatattgtaaaagataaatataatttttattaaaatttatatttattattattataagttcttagaaaatacattttgttttaaaataatactataaaaagaacaataataaaaataaaatacaattaaaacataattaaaaaagaaaaatacagacagtaaaaaaaatcgctatcccataaattacaaaacatgaattaataaacatatattgtttattaataaaattgtaagttatttttatatatacataataaattaaagatactgaaacaataattttaaatgtaaataaatgaataatgatatgttttatatgattcattattctatatttataaggaagattacaaaaaaaaaattcatatgtattttttttgtaatttctgtgtttatgtttttatttttataatatttttaatctattattaaataagcttttagccctcccacacataaccagagggcagcaattcacgaatcccaactgccgtcggctgtccatcactgtccttcactatcgctttgatcccaggatgcagatcgagaagcacctgtcgacaccgtccgcaggggctcaagatgcccctgttctcatttccgatcgcgacgatacaagtcaggttgccagctgccgcagcagcagcagtgcccagcaccacgagttctgcacaaggtcccccagtaaaatgatatacattgacaccagtgaagatgcggccgtcgctagagagagctgcgctggcgacgctgtagtcttcagagatggggatgctgttgattgtagccgttgctctttcaatgagggtggattcttcttgagacaaaggcatggatcctgatatatttctattaggtatttattattataaaatataaatcttgaatgataataaataaaatattagttattccttttctagtttaaaatatacatattataaatatatatatatatatatatatttttattgtgacaagaatatataattataaattatattatttatttttgtatttttttttttttttttttttttttctttttttgttttatttttctttttttttataaatattatttttttcttttatcatgcacattggaataatacattaatatatatatatatattatattatacatatattgaataatgtttataaaaaatgcataacttatatgaatataattttttttaaatatgacaaaaagaaaaaaaaaaaaaaccaaaaaaaattaaaattgaaatgaaatatataaatatattatttatatatattatacattgtttaatactactacatgtatatatatatattatatatatatatatatatcaattttttcaaaaataaattaatataaaaagaggggaaaaaaaaaaaaaaaaaaaaaaaaagataattaagtaagcatttaaaaatatataaattgataatatataaaattaatcacatataaactaatataatttataaaataaggaaaataaaatattaccataaaataaaaataaaaataaaaaaaaaaaaaaaaaacacctttttttatatatattaatatataattatctcttagaaaaaatattgtataattatatatgtaatgatttatataaaaaaataaaattatacaagtatatattttgtttctataaattgatacataacttaactaaggtcttacttttactaacttaggtcatacttctactaacataagtcttaacttacctaacataggtcttaacttgactaatctaggtatgaacttcaataactaaggtcttaacatgactaacataggtcttaacttaactaacttaggtcatacttcgattaactaaggtcttacattcactgatataggtcttaaggtacccatttaataataaggttattataaattttttttgtaacagttgtaaatatgtaggatgttcaaagtattctttttttgatattgtaagtattttattatatctagattcttacaataaatatattaaagctataggattaaattgtgtaaatatagaatatgtatatgatttattttatccctttcaaaaatatatatgtccgtataataatattaatactaatttgtatcacagtcaaaatattcaaatgaataacattgtaaaagatgtaatgaaggacctaaaaaaaaatagatatataaatgactttaattttttttgttcacctaataaatctttagaagtggtctcatttgatgatcaaaacggagatgtgcattttcataattcagcaaataagacaaatcatgtatacaactatattgaaaagtggataaatgtgcatgtaaatggttttggtgggtgttgtctgcaggatgaaaaatcccaaggaaaaaaaaaaaaaaatatgagcgaatattatcaacttgataaaaatattattaagaataaaaataatattgataggaatataaaaaaatctattaattataaagcatattataaatattctttatattatgatatacaaatgaatatatcaagtgttaattcttatggtcgagagaaatataatcctatagaaattgtacccaagatgaaaaaaaaaattcaaaatgtaaattgtactaataaggatgagacaaatataataaatacacacaataataataacaatgataacatattttttaataaaaaattatttaattatggaatagaatattatatagatgtaagtgatcaagagattatatcaaactgcctattcaaattaaataccttttgtaaaaacagtgacaaattacatttgttttctttaacaactttttcaaatataagagaagtattaacattttataattgtataaaatattatggatccaccttcaacaacaaagtcatcatcaacttcttctgcaattcatgcaagtacgttggatgtagtaaatactcattcttcgacatcgtttcaatcctgctgtacttagactcatataacaagtacatcaaggcaatcgggctgaactgcgttaacatcgagtacgtttacgacctattctacccattccagaagtacatctgcccatacaacaacatcaacacgaacttataccattcacagaacatccagatgaacaatatcgttaaggacgttatgaaagatttaaagaagaaccgctacatcaacgatttcaattttttctgctcgccaaacaagtcactggaggtagttagtttcgacgaccagaatggggacgttcacttccacaacagtgctaacaaaacgaaccacgtttataattacatcgagaaatggatcaacgttcacgttaacggattcggaggatgctgctactacaacccatatgacatctcattaatcaactataagatcaacgaactgctgtcag
